# Supplementary material for: Efficient Separation of Phytochemicals from Muehlenbeckia volcanica (Benth.) Endl. by Polarity-Stepwise Elution Counter-Current Chromatography and Their Antioxidant, Antiglycation, and Aldose Reductase Inhibition Potentials
Source: Molecules. 2021 Jan 4;26(1):224. doi: 10.3390/molecules26010224 (PMC7796107; doi:10.3390/molecules26010224)
Supplement: Supplementary file 1 [file molecules-26-00224-s001.zip › molecules-1054538-supplementary.pdf]

Supplementary Materials

Efficient Separation of Phytochemicals from *Muehlenbeckia volcanica* (Benth.) Endl. by Polarity-Stepwise Elution Counter-Current Chromatography and their Antioxidant, Antiglycation, and Aldose Reductase Inhibition Potentials

Guang-Lei Zuo [1], Hyun Yong Kim [1], Yanymee N. Guillen Quispe [1,2,3], Zhi-Qiang Wang [1,4], Seung Hwan Hwang [1,5], Kyong-Oh Shin [1] and Soon Sung Lim [1,6,7,*]

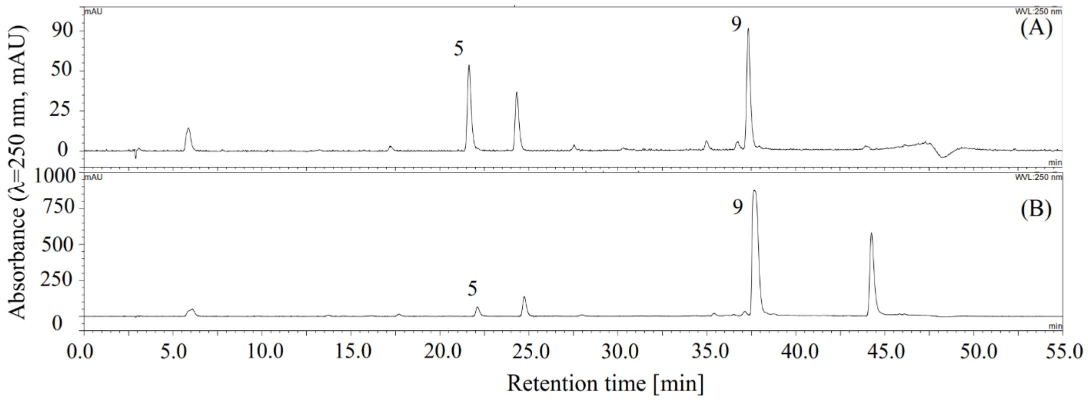

Figure S1. HPLC chromatograms of the compound **5**-containing mixture. (A) HPLC chromatogram of the freshly obtained compound **5**-containing CCC fraction. (B) Compound **5** degraded during preservation at 4 °C.

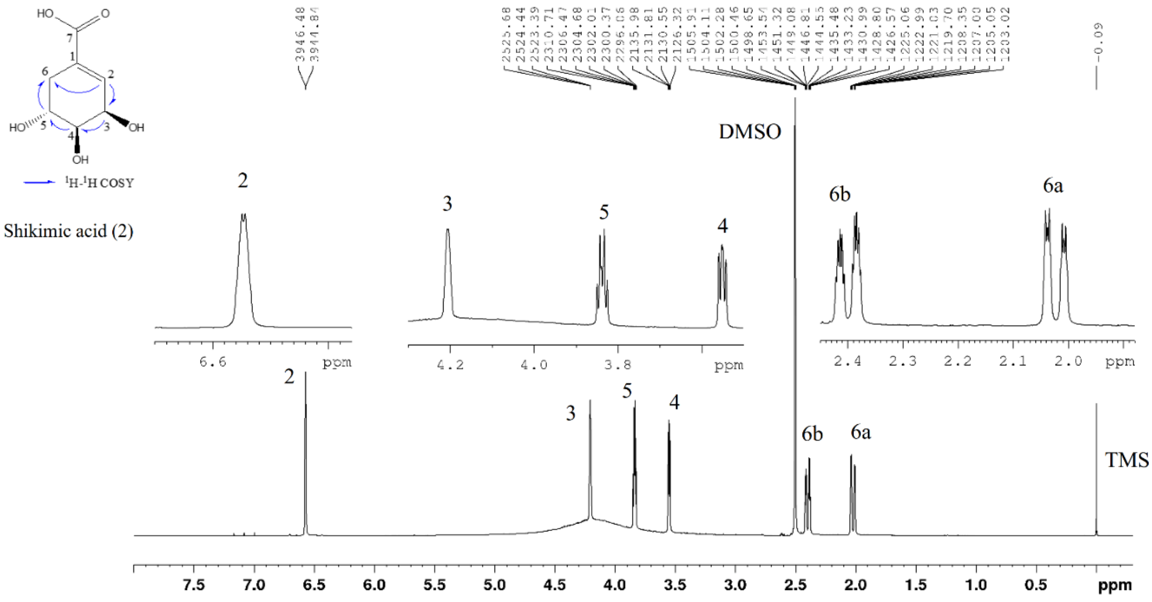

Figure S2-1. ^{1}H-NMR (600 MHz, DMSO-d_6) spectroscopy of shikimic acid (**2**) from plant *Muehlenbec kia volcanica* (Benth.) Endl.

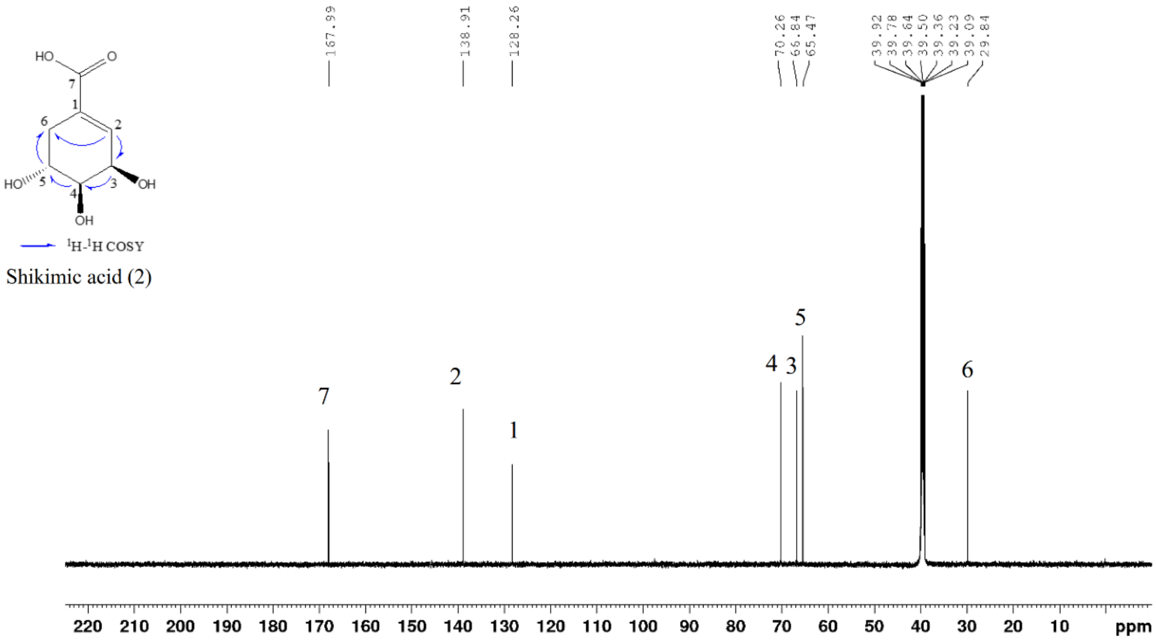

Figure S2-2. ^{13}C-NMR (150 MHz, DMSO-d_6) spectroscopy of shikimic acid (**2**) from plant *Muehlenbeckia volcanica* (Benth.) Endl.

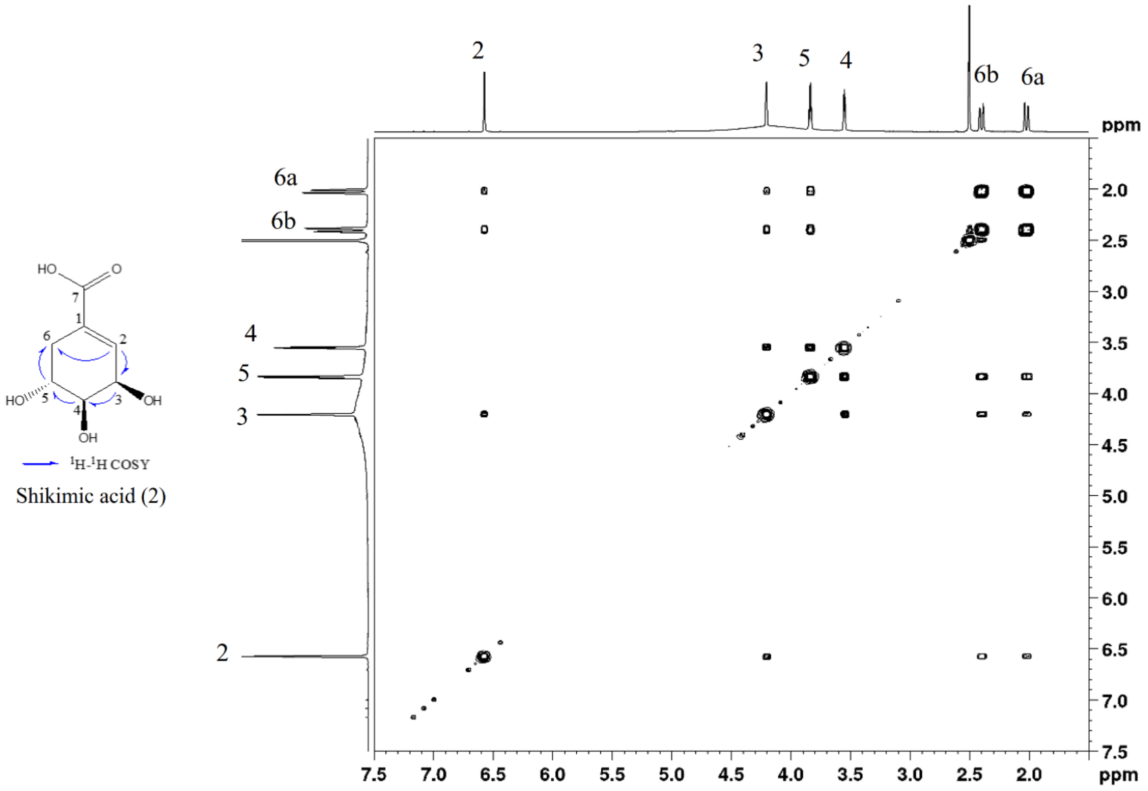

Figure S2-3. ^{1}H-^{1}H COSY NMR (600 MHz, DMSO-d_6) spectroscopy of shikimic acid (**2**) from plant *Muehlenbeckia volcanica* (Benth.) Endl.

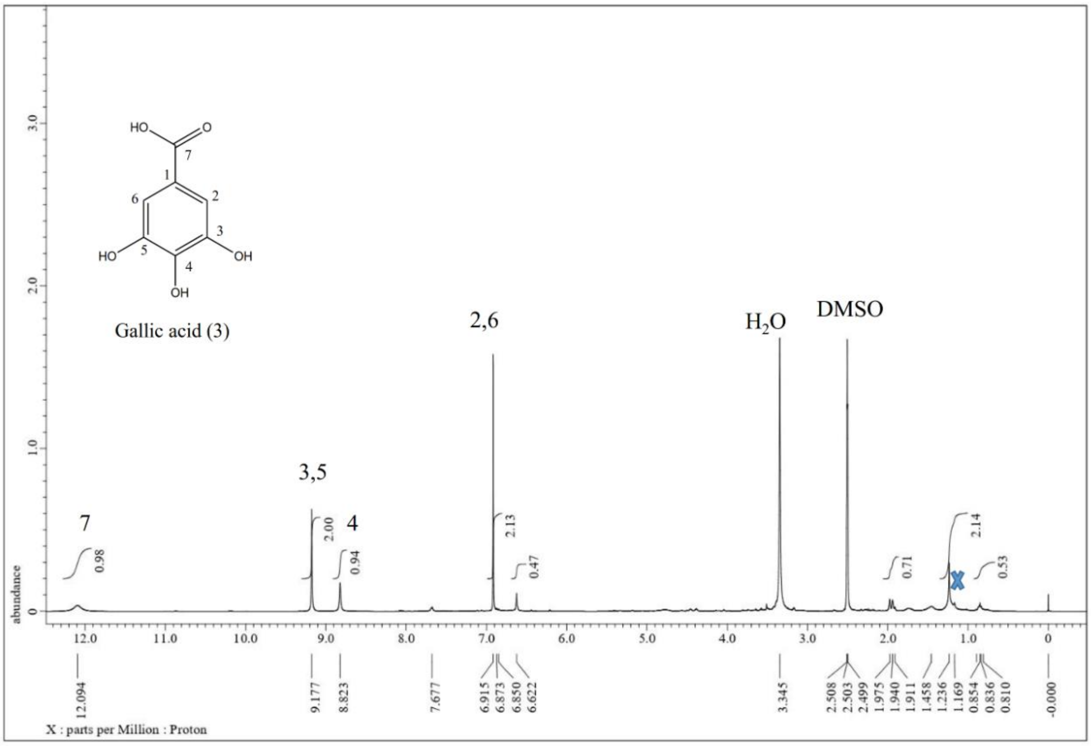

Figure S3. ^{1}H NMR (400 MHz, DMSO-d_6) spectroscopy of gallic acid (**3**) from plant *Muehlenbeckia v olcanica* (Benth.) Endl.

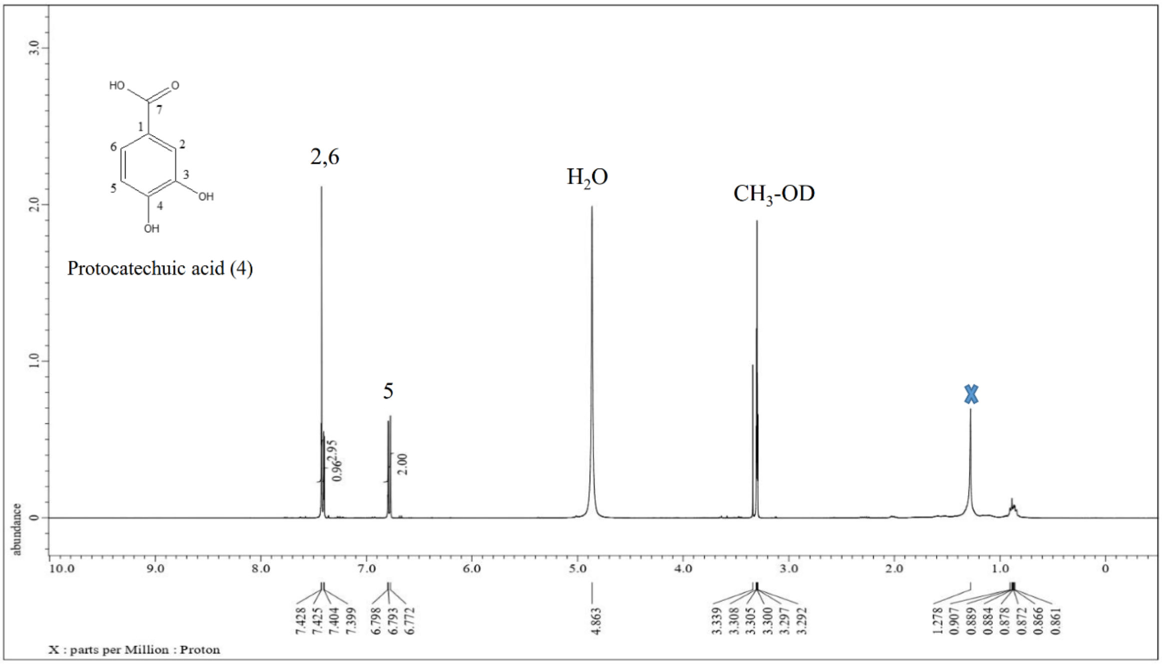

Figure S4-1. ^{1}H NMR (400 MHz, MeOD-d_4) spectroscopy of protocatechuic acid (**4**) from plant *Muehl enbeckia volcanica* (Benth.) Endl.

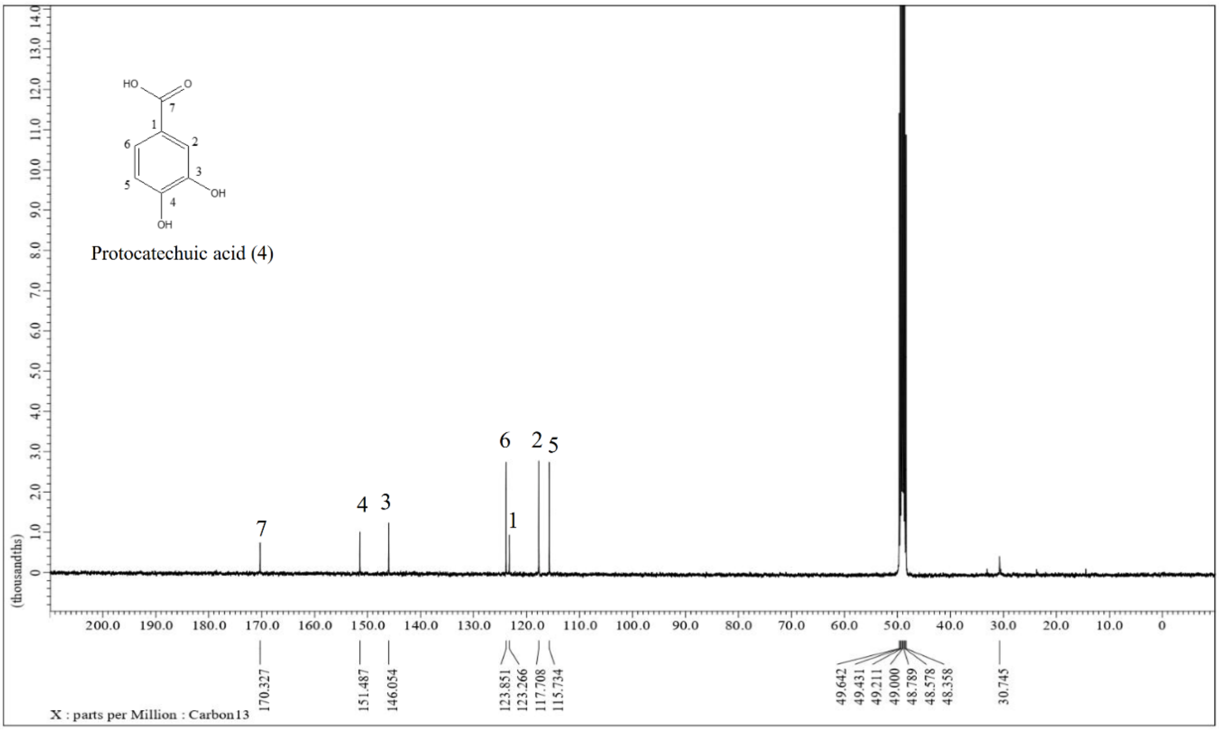

Figure S4-2. ^{13}C NMR (100 MHz, MeOD-d_4) spectroscopy of protocatechuic acid (**4**) from plant *Muehlenbeckia volcanica* (Benth.) Endl.

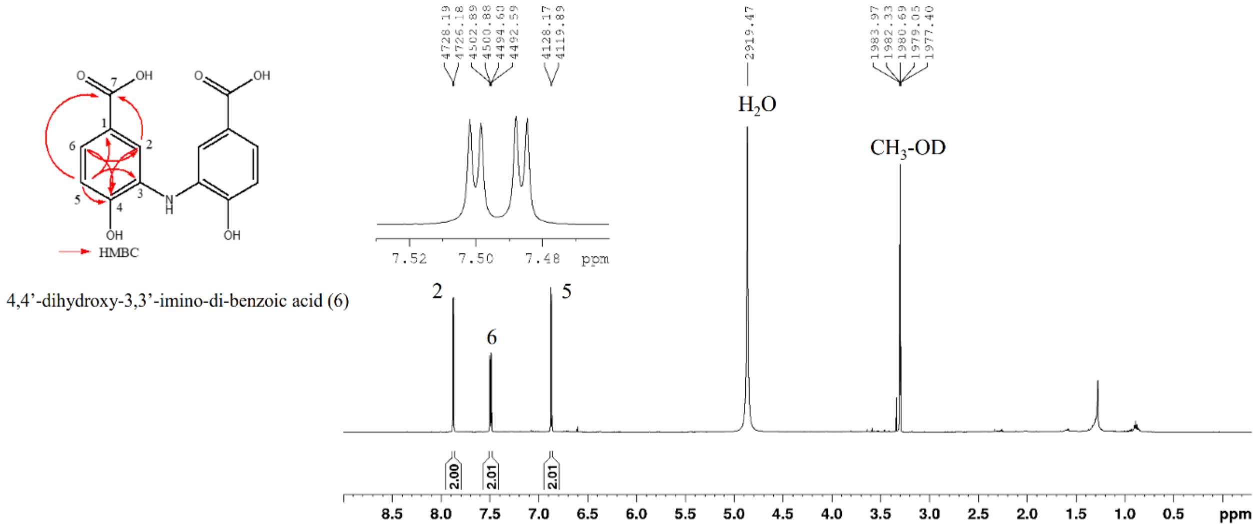

Figure S5-1. ^{1}H NMR (600 MHz, MeOD-d_4) spectroscopy of 4,4'-dihydroxy-3,3'-imino-di-benzoic acid (**6**) from plant *Muehlenbeckia volcanica* (Benth.) Endl.

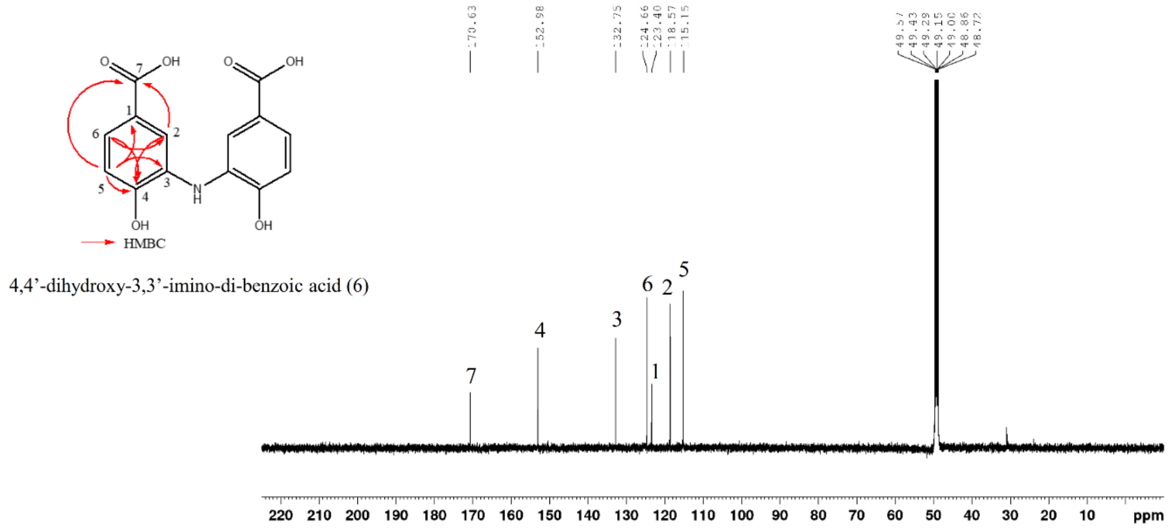

Figure S5-2. ^{13}C NMR (150 MHz, MeOD-d_4) spectroscopy of 4,4'-dihydroxy-3,3'-imino-di-benzoic acid (**6**) from plant *Muehlenbeckia volcanica* (Benth.) Endl.

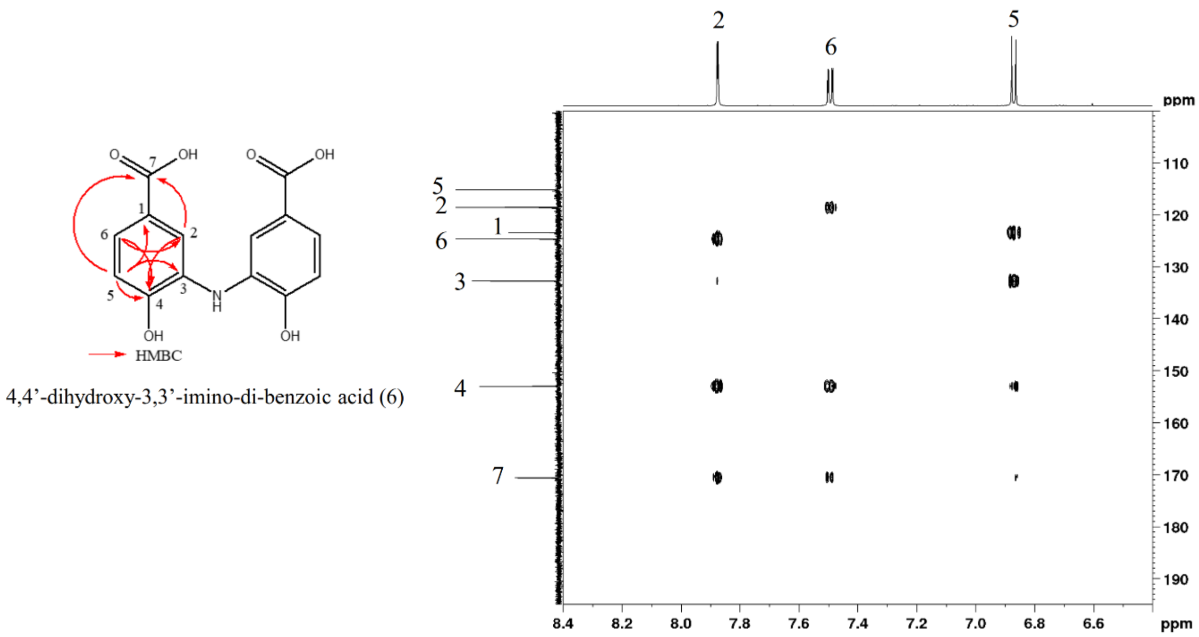

Figure S5-3. HMBC NMR spectroscopy of 4,4'-dihydroxy-3,3'-imino-di-benzoic acid (**6**) from plant *Mu ehlenbeckia volcanica* (Benth.) Endl.

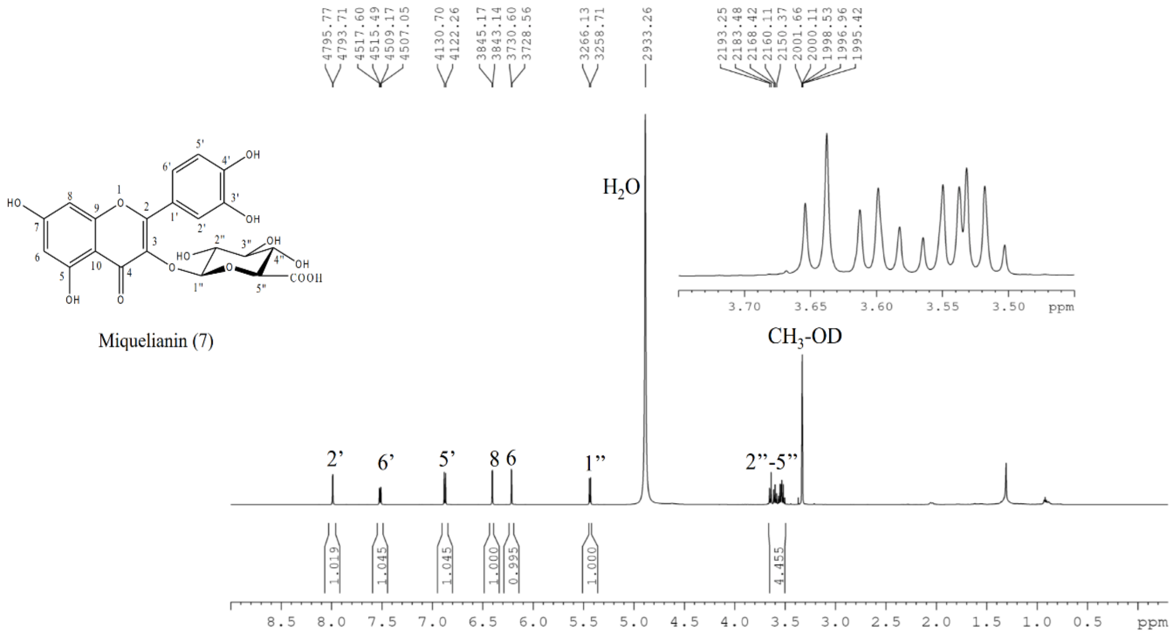

Figure S6. ^{1}H NMR (600 MHz, MeOD-d_4) spectroscopy of miquelianin (**7**) from plant *Muehlenbeckia volcanica* (Benth.) Endl.

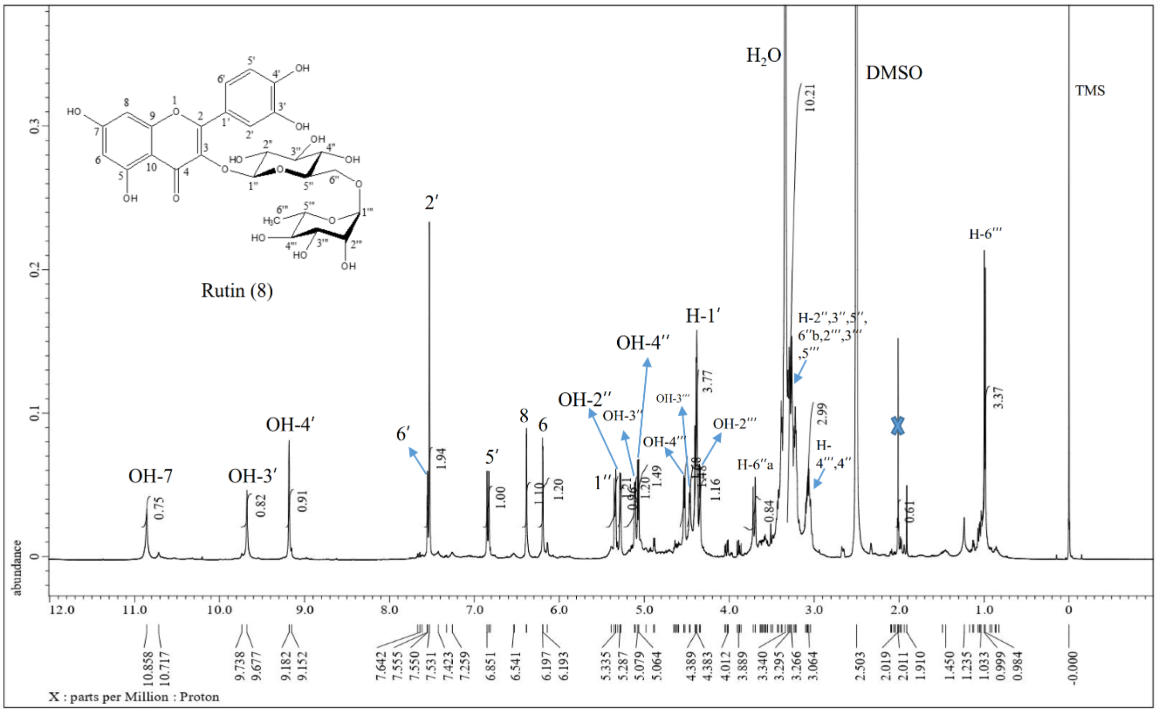

Figure S7. ^{1}H NMR (400 MHz, DMSO-d_6) spectroscopy of rutin (**8**) from plant *Muehlenbeckia volcani ca* (Benth.) Endl.

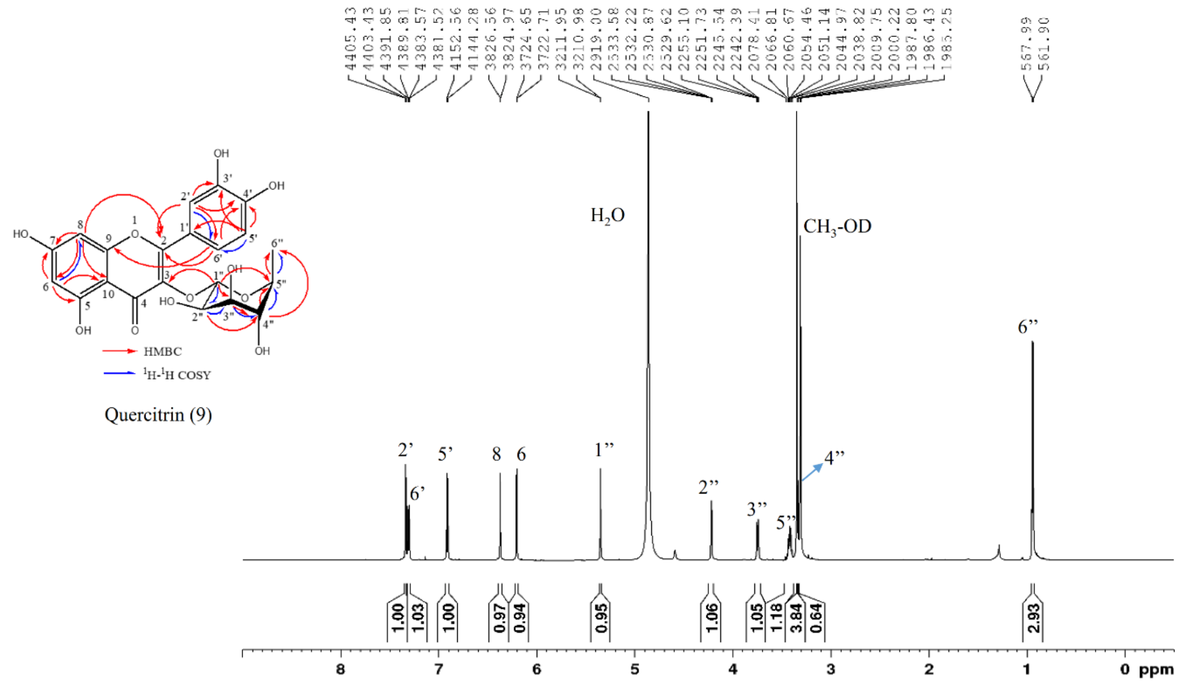

Figure S8-1. ^{1}H NMR (600 MHz, MeOD-*d*4) spectroscopy of quercitrin (**9**) from plant *Muehlenbeckia volcanica* (Benth.) Endl.

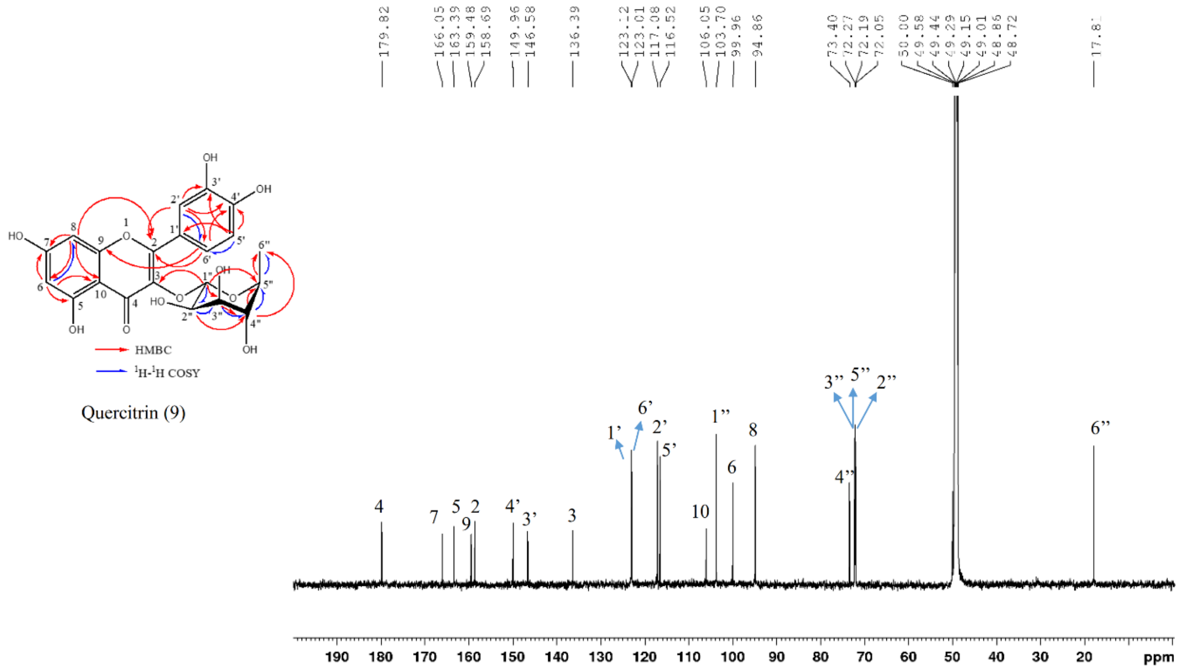

Figure S8-2. ^{13}C NMR (150 MHz, MeOD-*d*4) spectroscopy-1 of quercitrin (**9**) from plant *Muehlenbecki a volcanica* (Benth.) Endl.

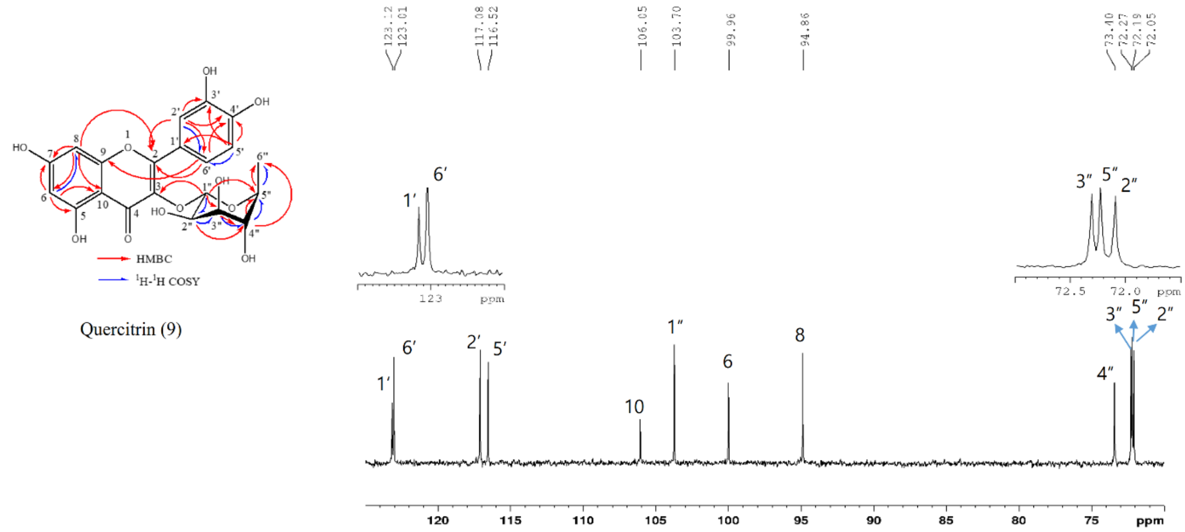

Figure S8-3. ^{13}C NMR (150 MHz, MeOD-d_4) spectroscopy-2 of quercitrin (**9**) from plant *Muehlenbecki a volcanica* (Benth.) Endl.

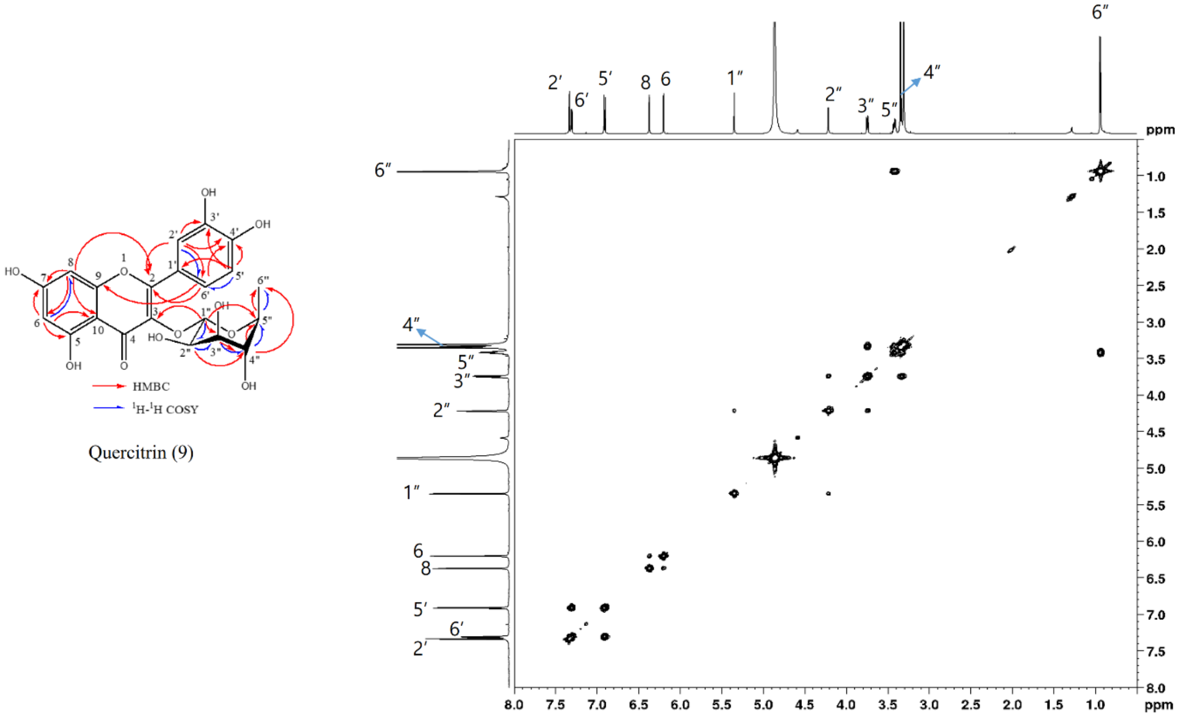

Figure S8-4. ^{1}H-^{1}H COSY NMR (600 MHz, MeOD-d_4) spectroscopy of quercitrin (**9**) from plant *Mueh lenbeckia volcanica* (Benth.) Endl.

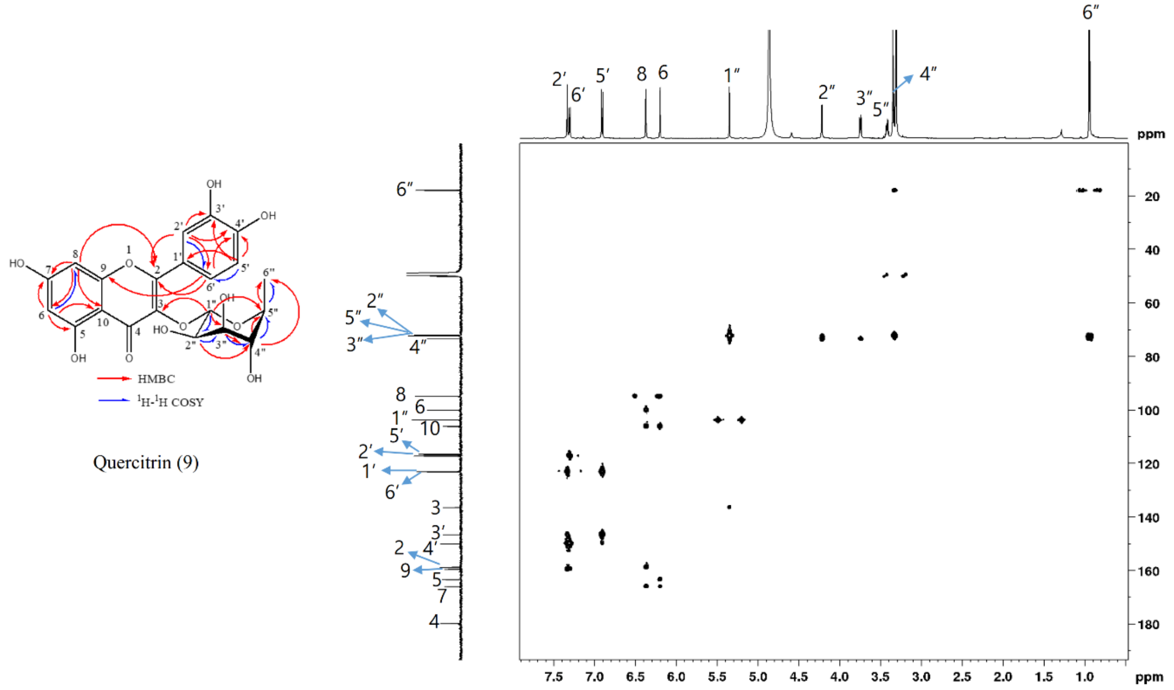

Figure S8-5. HMBC NMR spectroscopy of quercitrin (**9**) from plant *Muehlenbeckia volcanica* (Benth.) Endl.

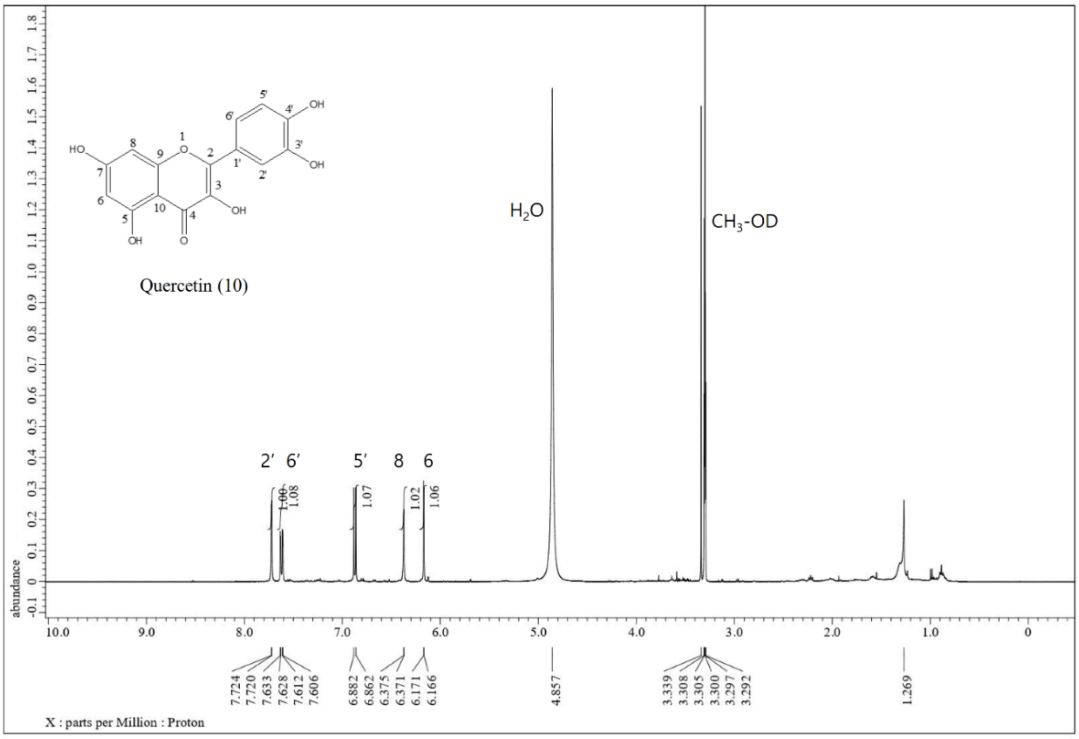

Figure S9. ^{1}H NMR (400 MHz, MeOD-d_4) spectroscopy of quercetin (**10**) from plant *Muehlenbeckia v olcanica* (Benth.) Endl.

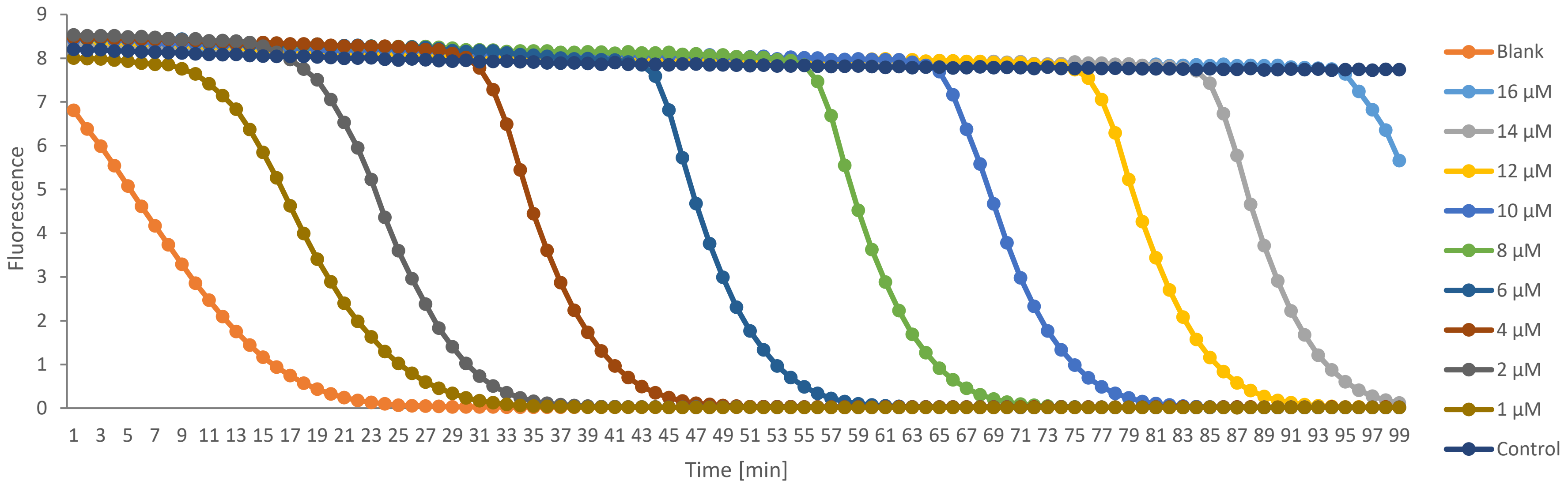

Figure S10-1. Fluorescein fluorescence decay cure induced by AAPH in the presence of trolox, 1-16 µM, or in the absence of trolox (Blank group). Where as Control group represents the fluorescein fluorescence decay cure without addition of AAPH or trolox.

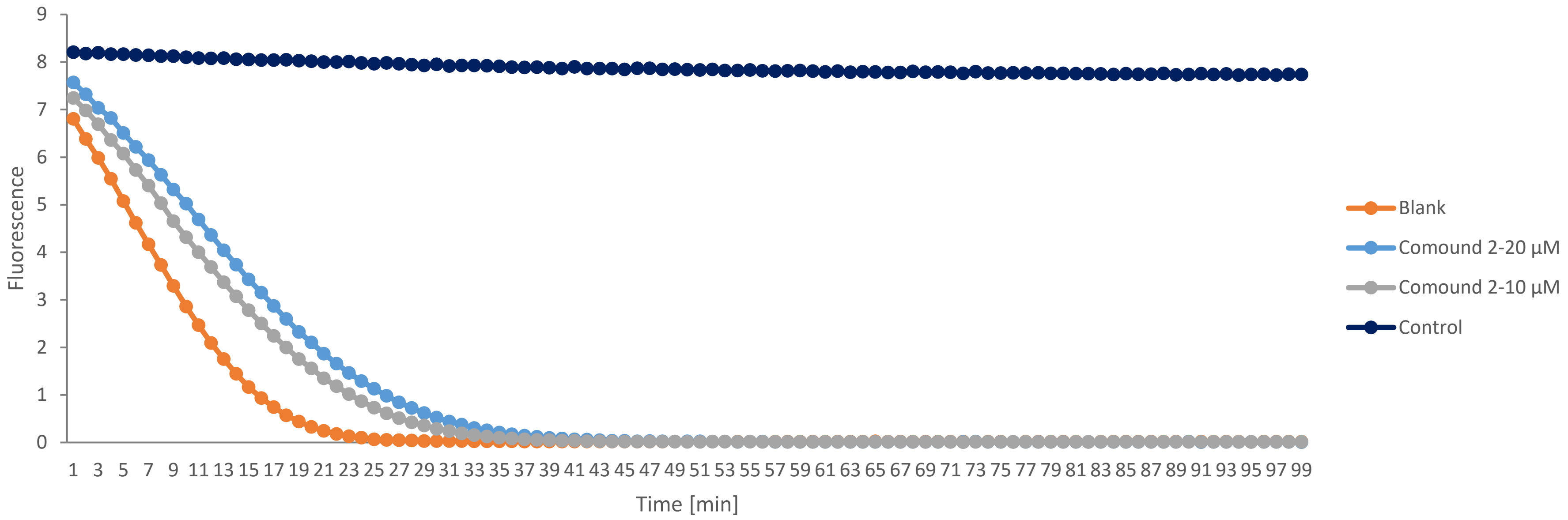

Figure S10-2. Fluorescein fluorescence decay cure induced by AAPH in the presence of shikimic acid (compound **2**, 10 and 20 µM), or in the absence of antioxidants (Blank group). Whereas Control group represents the fluorescein fluorescence decay cure without addition of AAPH or antioxidants.

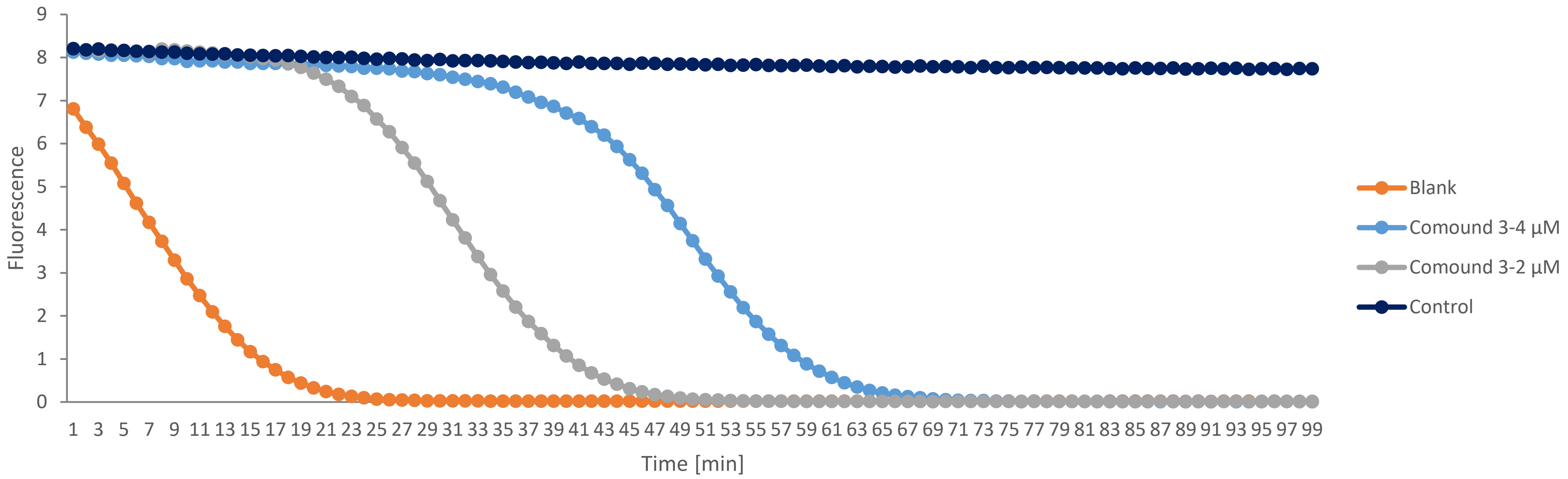

Figure S10-3. Fluorescein fluorescence decay cure induced by AAPH in the presence of gallic acid (compound **3**, 2 and 4 μM), or in the absence of antiox idants (Blank group). Whereas Control group represents the fluorescein fluorescence decay cure without addition of AAPH or antioxidants.

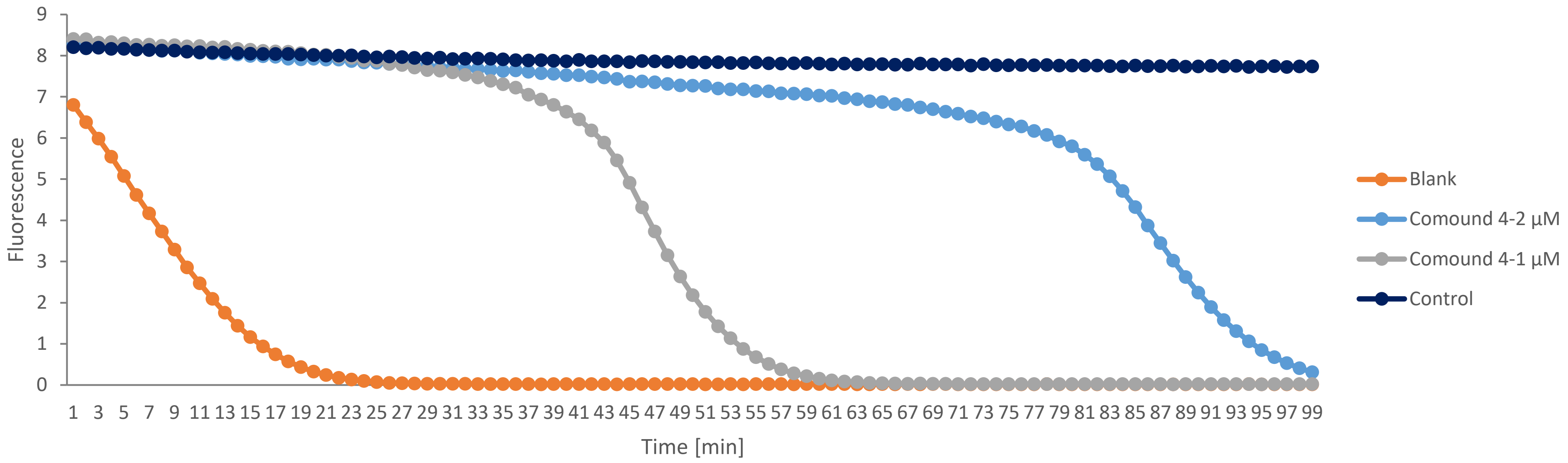

Figure S10-4. Fluorescein fluorescence decay cure induced by AAPH in the presence of protocatechuic acid (compound **4**, 1 and 2 μM), or in the absence of antioxidants (Blank group). Whereas Control group represents the fluorescein fluorescence decay cure without addition of AAPH or antioxidants.

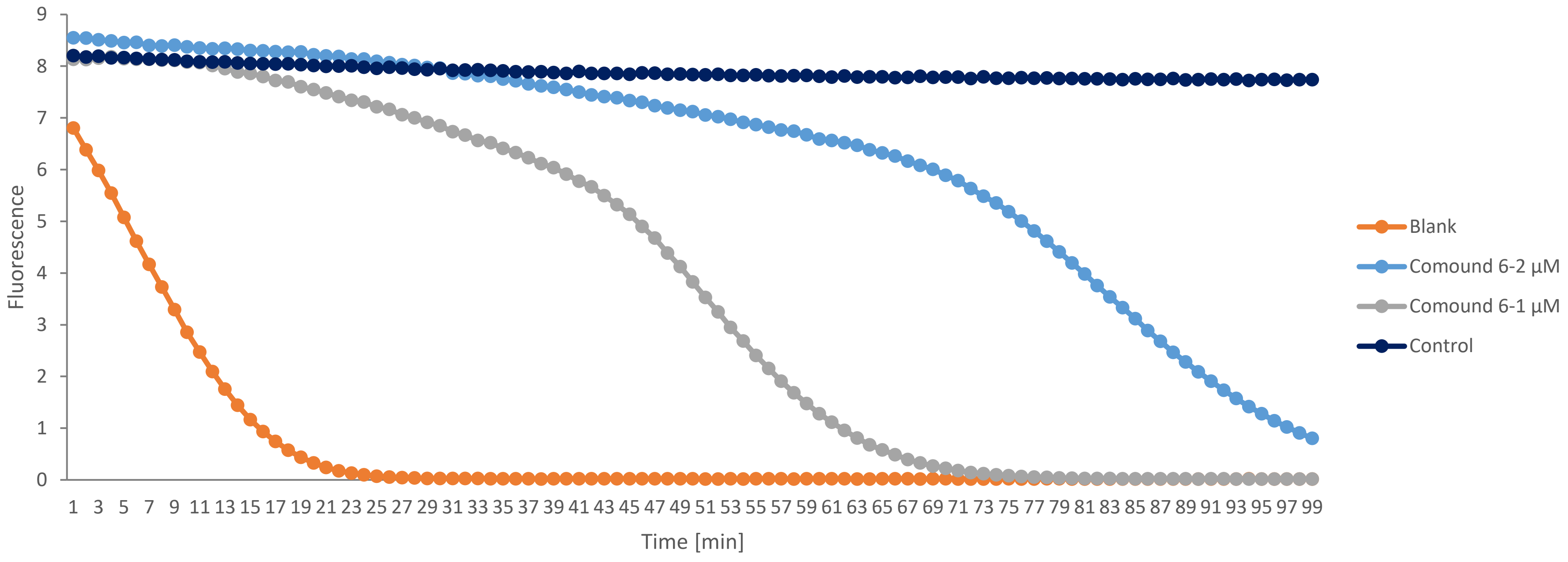

Figure S10-5. Fluorescein fluorescence decay cure induced by AAPH in the presence of 4,4'-dihydroxy-3,3'-imino-di-benzoic acid (compound **6**, 1 and 2 μM), or in the absence of antioxidants (Blank group). Whereas Control group represents the fluorescein fluorescence decay cure without addition of AAPH or antioxidants.

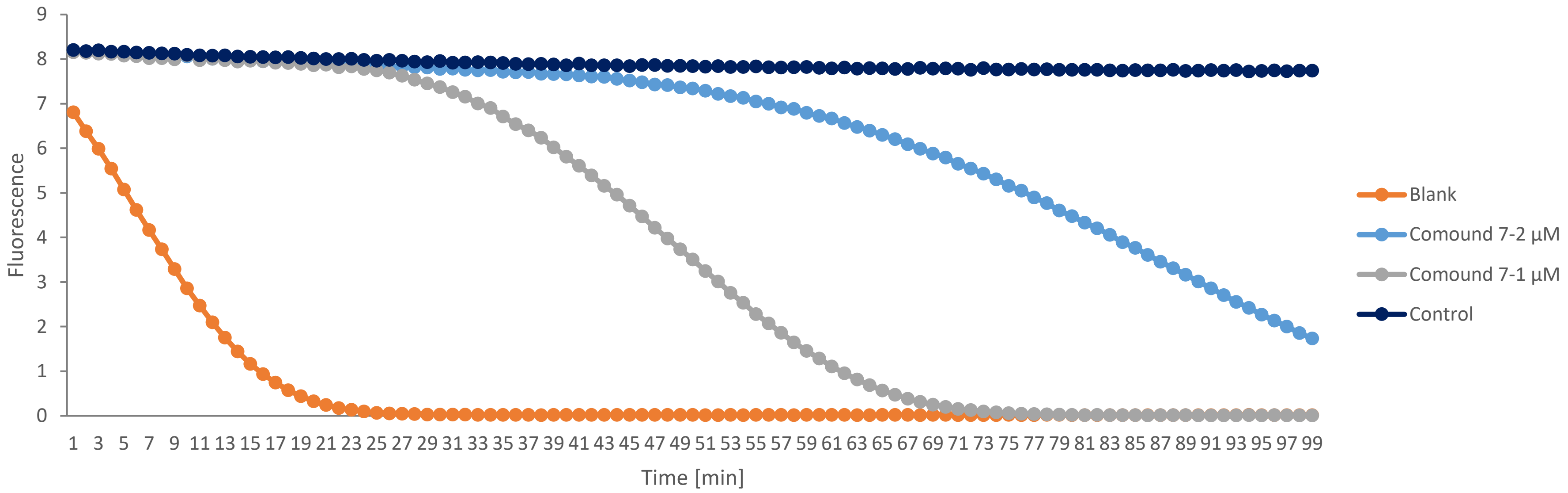

Figure S10-6. Fluorescein fluorescence decay cure induced by AAPH in the presence of miquelianin (compound **7**, 1 and 2 μM), or in the absence of antio xidants (Blank group). Whereas Control group represents the fluorescein fluorescence decay cure without addition of AAPH or antioxidants.

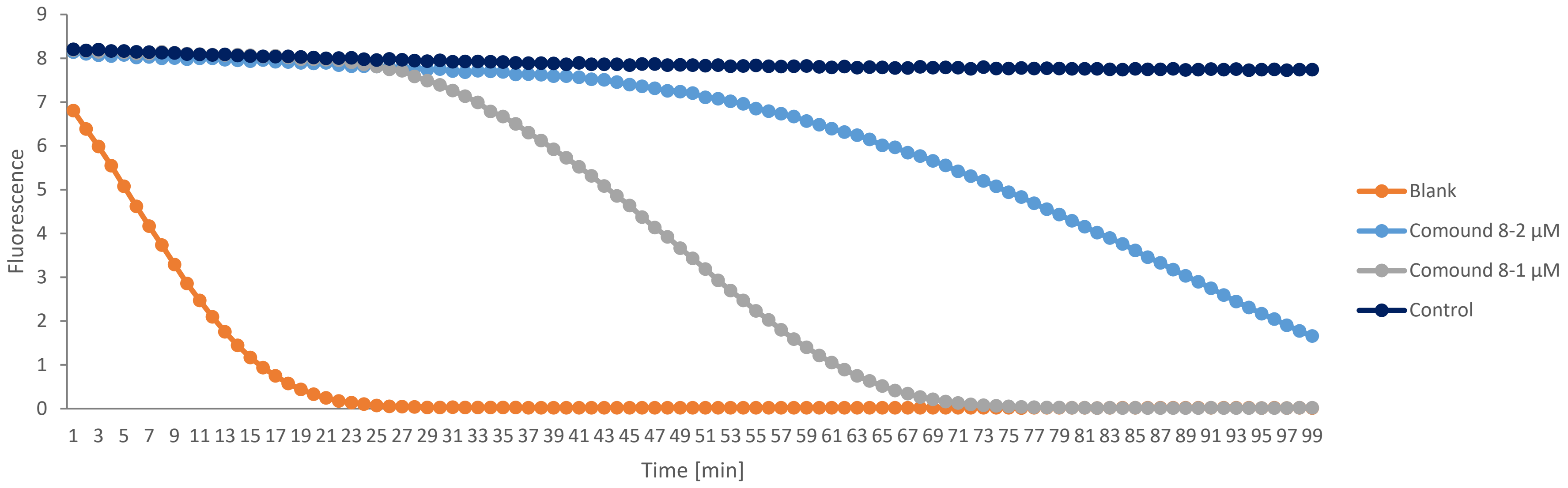

Figure S10-7. Fluorescein fluorescence decay cure induced by AAPH in the presence of rutin (compound **8**, 1 and 2 μM), or in the absence of antioxidants (Blank group). Whereas Control group represents the fluorescein fluorescence decay cure without addition of AAPH or antioxidants.

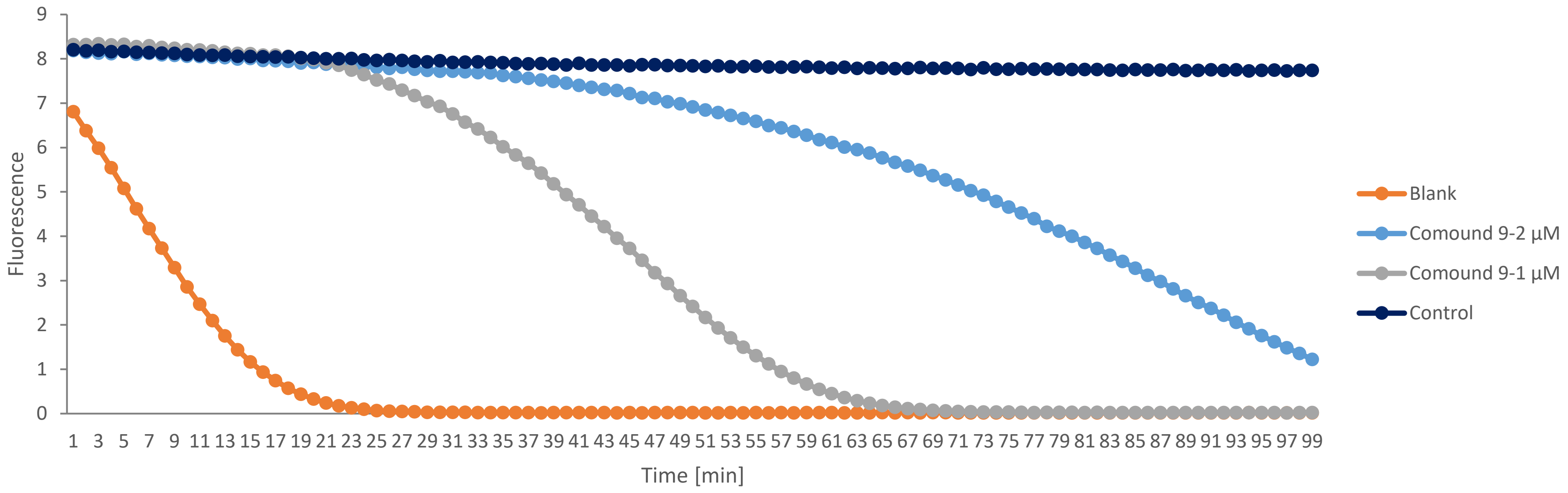

Figure S10-8. Fluorescein fluorescence decay cure induced by AAPH in the presence of quercitrin (compound **9**, 1 and 2 μM), or in the absence of antioxi dants (Blank group). Whereas Control group represents the fluorescein fluorescence decay cure without addition of AAPH or antioxidants.

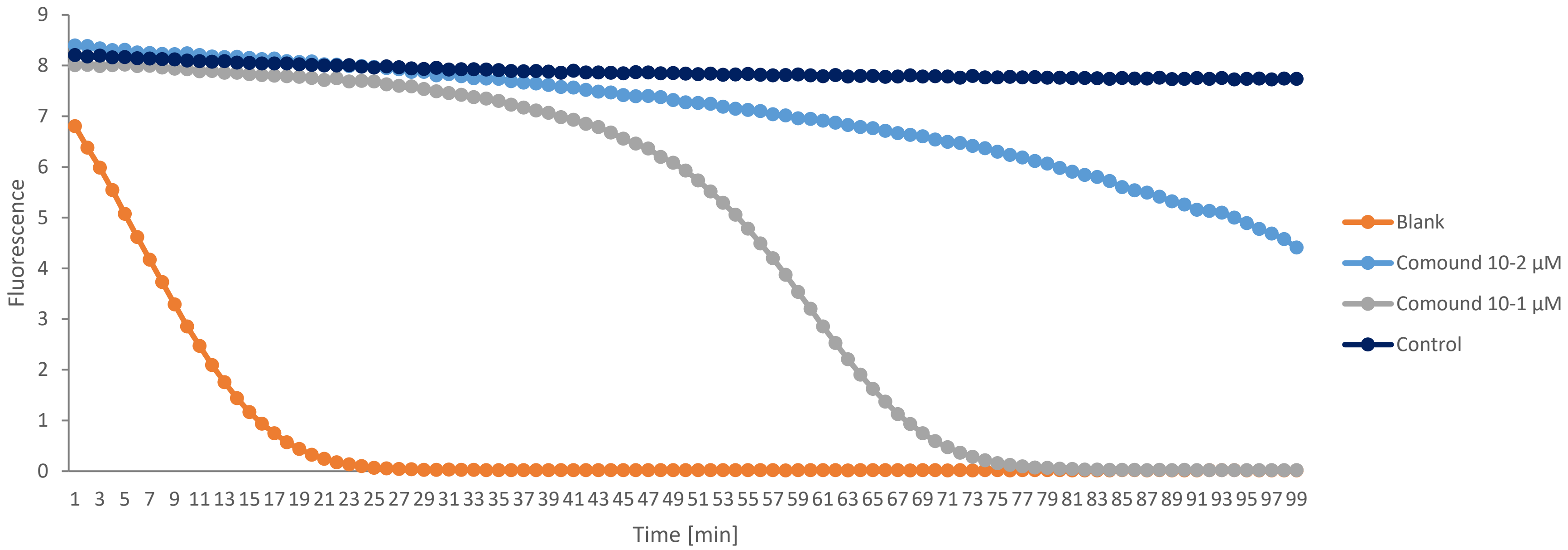

Figure S10-9. Fluorescein fluorescence decay cure induced by AAPH in the presence of quercetin (compound **10**, 1 and 2 μM), or in the absence of antiox idants (Blank group). Whereas Control group represents the fluorescein fluorescence decay cure without addition of AAPH or antioxidants.

Table S1. Partition coefficients ($K_{upper/lower}$) of compounds **1-10** from the 70% methanol extract of *Muehlenbeckia volcanica* (Benth.) Endl. at a serious of *n*-h exane/ethyl acetate/methanol/*n*-butanol/water solvent systems.

Solvent system	*K* values								
	1&2	3	4	5	6	7	8	9	10
n-Hexane/EA/MeOH/water 4:5:4:5, v/v	0.02	0.02	0.09	0.01	0.04	0.00	0.00	0.02	**0.78**
n-Hexane/EA/MeOH/water 3:5:3:5, v/v	0.02	0.09	0.29	0.07	0.27	0.01	0.01	0.12	2.91
n-Hexane/EA/MeOH/water 2:5:2:5, v/v	0.02	0.18	**0.75**	0.33	**1.12**	0.04	0.03	0.45	8.76

n-Hexane/EA/MeOH/water 1:5:1:5, v/v	0.02	0.44	1.62	**1.18**	4.58	0.11	0.10	**1.21**	8.14
EA/water 5:5, v/v	0.05	**1.33**	3.67	5.31	16.34	0.28	0.31	3.40	15.19
EA/*n*-BuOH/water 4:1:5, v/v	0.07	2.79	8.32	32.90	29.05	**1.42**	**1.50**	12.92	32.48
EA/*n*-BuOH/water 3:2:5, v/v	0.09	5.22	9.32	70.27	54.42	3.57	3.87	22.26	44.55
EA/*n*-BuOH/water 2:3:5, v/v	0.20	6.58	8.65	41.31	54.04	4.36	5.33	15.94	31.91
n-BuOH/water 5:5, v/v	0.32	5.84	8.51	40.91	32.41	5.44	6.65	12.77	29.64

EA: ethyl acetate; MeOH: methanol; water: ultrapure water; *n*-BuOH: *n*-butanol. All *n*-BuOH used in this study has been pre-saturated using ultrapure water.

Table S2. Structural assignment of the separated compounds from the 70% methanol extract of *Muehlenbeckia vulcanica* Meisn. using NMR, MS, and UV analysis.

Peak number	UV (λmax, nm) by HPLC	EI-MS *m/z* (abundance)	LC-ESI-MS *m/z*	Formula (molecular weight)	^{1}H NMR, δ (ppm)	^{13}C NMR, δ (ppm)	Structural assignment [a]
2	-[b]	-	negative ion: 172.6 [M-H]$^-$	$C_7H_{10}O_5$ (174)	600 MHz (DMSO-d_6) δ_H: 6.57 (1H, d, *J*= 1.64 Hz, 2-H), 4.20-4.21 (1H, m, 3-H), 3.84 (1H, dd, *J*= 10.40 Hz, 4.27 Hz, 5-H), 3.55 (1H, dd, *J*= 5.43 Hz, 4.20 Hz, 4-H), 2.40 (1H, ddt, *J*= 18.01 Hz, 4.52 Hz, 2.22 Hz, 6a-H), 2.02 (1H, ddt, *J*= 18.06 Hz, 4.03 Hz, 2.03 Hz, 6b-H).	150 MHz (DMSO-d_6) δ_C: 168.0 (C-7), 138.9 (C-2), 128.3 (C-1), 70.3 (C-4), 66.8 (C-5), 65.5 (C-3), 29.8 (C-6).	Shikimic acid
3	234, 270	170 (100.00%), 153 (99.57%), 125 (54.07%)	negative ion: 169.1 [M-H]$^-$	$C_7H_6O_5$ (170)	400 MHz (DMSO-d_6) δ_H: 12.10 (1H, br s, COOH), 9.18 (2H, s, 2-OH), 8.82 (1H, s, 3-OH), 6.92 (2H, s, 1-H).	-	Gallic acid
4	258, 294	154 (95%), 137 (96%), 109 (100%), 81 (38%).	negative ion: 153.0 [M-H]$^-$	$C_7H_6O_4$ (154)	400 MHz (MeOD-d_4) δ_H: 7.43 (1H, d, *J*= 2.4 Hz, 2-H), 7.41 (1H, dd, *J*= 8.8 Hz, 2.0 Hz, 6-H), 6.78 (1H, d, *J*= 8.4 Hz, 5-H).	100 MHz (DMSO-d_6) δ_C: 170.33 (C-7), 151.49(C-4), 146.05 (C-3), 123.85 (C-6), 123.27 (C-1), 117.71 (C-2), 115.73 (C-5).	Protocatechuic acid

6	249, 295, 379	289 (12%), 245 (100%), 227 (59%), 199 (32%), 107 (48%)	HRESI-MS (TOF, positive ion) *m/z*: 290.0663 $[M+H]^+$ (calculated for $C_{14}H_{12}NO_6^+$ 290.0665); 312.0487 $[M+Na]^+$, (calculated for $C_{14}H_{11}NO_6Na^+$ 312.0484).	$C_{14}H_{11}NO_6$ (289)	600 MHz (MeOD-d_4) δ_H: 7.88 (2H, d, *J*= 2.0 Hz, 2, 2'-H), 7.49 (2H, dd, *J*=8.3 Hz, 2.0 Hz, 6, 6'-H), 6.87 (2H, d, *J*= 8.3 Hz, 5, 5'-H).	150 MHz (MeOD-d_4) δ_C: 170.63 (C-7), 152.98 (C-4), 132.75 (C-3), 124.66 (C-6), 123.40 (C-1), 118.57 (C-2), 115.15 (C-5).	4,4'-dihydroxy-3,3'-imino-di-benzoic acid
7	255, 356	-	negative ion: 477.2 $[M-H]^-$.	$C_{21}H_{18}O_{13}$ (478)	600 MHz (MeOD-d_4) δ_H: 7.99 (1H, d, *J*= 2.1 Hz, 2'-H), 7.52 (1H, dd, *J*= 8.4 Hz, 2.1 Hz, 6'-H), 6.88 (1H, d, *J*= 8.4 Hz, 5'-H), 6.40 (1H, d, *J*= 2.0 Hz, 8-H), 6.21 (1H, d, *J*= 2.0 Hz, 6-H), 5.44 (1H, d, *J*= 7.4 Hz, 1''-H), 3.58-3.65 (4H, m, 2''-H, 3''-H, 4''-H, 5''-H).	-	Miquelianin
8	255, 356	-	negative ion: 608.9 $[M-H]^-$.	$C_{27}H_{30}O_{16}$ (610.5)	400 MHz (DMSO-d_6) δ_H: 10.86 (1H, br s, 7-OH), 9.68 (1H, br s, 3'-OH), 9.18 (1H, br s, 4'-OH), 7.56 (1H, dd, *J*= 8.4 Hz, 2.0 Hz, 6'-H), 7.55 (1H, d, *J*= 2.0 Hz, 2'-H), 6.84 (1H, d, *J*= 8.4 Hz, 5'-H), 6.39 (1H, d, *J*= 2.0 Hz, 8-H), 6.19 (1H, d, *J*= 2.0 Hz, 6-H), 5.34 (1H, d, *J*= 7.2 Hz, 1''-H), 5.28 (1H, d, *J*= 3.6 Hz, 2''-OH), 5.11 (1H, d, *J*= 3.6 Hz, 3''-OH), 5.07 (1H, d, *J*= 6.0 Hz, 4''-OH), 4.53 (1H, d, *J*= 5.2 Hz, 4'''-OH), 4.46 (1H, d, *J*= 4.4 Hz, 3'''-OH), 4.34-4.40 (2H, m, 1'''-H, 2'''-OH), 3.71 (1H, d, *J*= 10 Hz, 6''a-H), 3.22-3.39 (7H, m, 2''H, 3''H, 5''H, 6''bH, 2'''-H, 3'''-H. 5'''-H), 3.04-3.09 (2H, m, 4'''-H, 4''-H), 1.02 (3H, d, *J*= 6.6 Hz, 6'''-H).	-	Rutin

9	254, 352	-	negative ion: 447.1 [M-H]$^-$.	$C_{21}H_{20}O_{11}$ (448)	600 MHz (MeOD-d_4) δ_H: 7.34 (1H, d, *J*= 2.0 Hz, 2'-H), 7.31 (1H, dd, *J*= 8.3 Hz, 2.0 Hz, 6'-H), 6.91 (1H, d, *J*= 8.2 Hz, 5'-H), 6.37 (1H, d, *J*= 1.7 Hz, 8-H), 6.20 (1H, d, *J*= 1.9 Hz, 6-H), 5.35 (1H, d, *J*= 1.0 Hz, 1"-H), 4.22 (1H, dd, *J*= 2.7 Hz, 1.4 Hz, 2"-H), 3.75 (1H, d, *J*= 9.3 Hz, 3.1 Hz, 3"-H), 3.40-3.46 (1H, m, 5"-H), 3.33 (1H, d, *J*= 9.5 Hz, 4"-H), 0.94 (3H, d, *J*= 6.1 Hz, 6"-H).	150 MHz (MeOD-d_4) δ_C: 179.82 (C-4), 166.05 (C-7), 163.39 (C-5), 159.48 (C-5), 158.69 (C-2), 149.96 (C-4'), 146.58 (C-3'), 136.39 (C-3), 123.12 (C-1'), 123.01 (C-6'), 117.08 (C-2'), 116.52 (C-5'), 106.05 (C-10), 103.70 (C-1"), 99.96 (C-6), 94.86 (C-8), 73.40 (C-4"), 72.27 (C-3"), 72.19 (C-5"), 72.05 (C-2"), 17.81 (C-6").	Quercitrin
10	255, 306, 368	302 (100%), 301 (43%), 274 (13%), 273 (17%), 245(10%), 137 (18%)	negative ion: 300.9 [M-H]$^-$.	$C_{15}H_{10}O_7$ (302)	400 MHz (MeOD-d_4) δ_H: 7.72 (1H, d, *J*= 1.6 Hz, 2'-H), 7.62 (1H, d, *J*= 8.4 Hz, 2.0 Hz, 6'-H), 6.87 (1H, d, *J*= 8 Hz, 5'-H), 6.37 (1H, d, *J*= 1.6 Hz, 8-H), 6.17 (1H, d, *J*= 2.0 Hz).	-	Quercetin

[a] The related references are shown in the manuscript in Section 3.4.

[b] Data not chekced.

Table S3. Calibration cures of DPPH and ABTS radical inhibition (%) or AUC value by Trolox.

Antioxidant assay	Calibration curves of radical inhibition (%) by Trolox[a]		
	Linearity range of Ttrolox (μM)	Progression	r^2
DPPH	6.25-100	y = 0.7456x + 3.6613	0.9999
ABTS	0.52-16.67	y = 5.8398x -1.0452	0.9998
ORAC	2.00-16.00	y = 5.7544x + 10.948	0.9939

[a] The calibration cures were created by plotting DPPH and ABTS radical inhibition (%) or net AUC value (ORAC assay) against Trolox concentrations.
